# Exploring the effects of extended interval dosing of natalizumab and drug concentrations on brain atrophy in multiple sclerosis

**DOI:** 10.1177/13524585231225855

**Published:** 2024-01-18

**Authors:** Alyssa A Toorop, Samantha Noteboom, Martijn D Steenwijk, Job W Gravendeel, Bas Jasperse, Frederik Barkhof, Eva MM Strijbis, Theo Rispens, Menno M Schoonheim, Zoé LE van Kempen, Joep Killestein

**Affiliations:** MS Center Amsterdam, Department of Neurology, Amsterdam Neuroscience, Vrije Universiteit Amsterdam, Amsterdam UMC, Amsterdam, The Netherlands; MS Center Amsterdam, Department of Anatomy & Neurosciences, Amsterdam Neuroscience, Vrije Universiteit Amsterdam, Amsterdam UMC, Amsterdam, The Netherlands; MS Center Amsterdam, Department of Anatomy & Neurosciences, Amsterdam Neuroscience, Vrije Universiteit Amsterdam, Amsterdam UMC, Amsterdam, The Netherlands; MS Center Amsterdam, Department of Neurology, Amsterdam Neuroscience, Vrije Universiteit Amsterdam, Amsterdam UMC, Amsterdam, The Netherlands; MS Center Amsterdam, Department of Radiology and Nuclear Medicine, Amsterdam Neuroscience, Vrije Universiteit Amsterdam, Amsterdam UMC, Amsterdam, The Netherlands; MS Center Amsterdam, Department of Radiology and Nuclear Medicine, Amsterdam Neuroscience, Vrije Universiteit Amsterdam, Amsterdam UMC, Amsterdam, The Netherlands; Queen Square Institute of Neurology and Centre for Medical Image Computing, University College London, London, UK; MS Center Amsterdam, Department of Neurology, Amsterdam Neuroscience, Vrije Universiteit Amsterdam, Amsterdam UMC, Amsterdam, The Netherlands; Biologics Laboratory and Department of Immunopathology, Sanquin Diagnostic Services, Amsterdam, The Netherlands; Landsteiner Laboratory, Academic Medical Center, Amsterdam UMC, University of Amsterdam, Amsterdam, The Netherlands; MS Center Amsterdam, Department of Anatomy & Neurosciences, Amsterdam Neuroscience, Vrije Universiteit Amsterdam, Amsterdam UMC, Amsterdam, The Netherlands; MS Center Amsterdam, Department of Neurology, Amsterdam Neuroscience, Vrije Universiteit Amsterdam, Amsterdam UMC, Amsterdam, The Netherlands; MS Center Amsterdam, Department of Neurology, Amsterdam Neuroscience, Vrije Universiteit Amsterdam, Amsterdam UMC, Amsterdam, The Netherlands

**Keywords:** Multiple sclerosis, natalizumab, brain atrophy, extended interval dosing, drug concentration

## Abstract

**Background:**

Extended interval dosing (EID) of natalizumab treatment is increasingly used in multiple sclerosis. Besides the clear anti-inflammatory effect, natalizumab is considered to have neuroprotective properties as well.

**Objectives:**

This study aimed to study the longitudinal effects of EID compared to standard interval dosing (SID) and natalizumab drug concentrations on brain atrophy.

**Methods:**

Patients receiving EID or SID of natalizumab with a minimum radiological follow-up of 2 years were included. Changes in brain atrophy measures over time were derived from clinical routine 3D-Fluid Attenuated Inversion Recovery (FLAIR)-weighted magnetic resonance imaging (MRI) scans using SynthSeg.

**Results:**

We found no differences between EID (*n* = 32) and SID (*n* = 50) for whole brain (−0.21% vs −0.16%, *p* = 0.42), ventricular (1.84% vs 1.13%, *p* = 0.24), and thalamic (−0.32% vs −0.32%, *p* = 0.97) annualized volume change over a median follow-up of 3.2 years. No associations between natalizumab drug concentration and brain atrophy rate were found.

**Conclusion:**

We found no clear evidence that EID compared to SID or lower natalizumab drug concentrations have a negative impact on the development of brain atrophy over time.

## Introduction

Natalizumab is a monoclonal antibody used for the treatment of relapsing–remitting multiple sclerosis (RRMS).^1^ Despite effective suppression of MS disease activity, disease progression and neurodegeneration still occur during natalizumab treatment.^2,3^ Brain atrophy seems slowed by natalizumab treatment.^4^ However, atrophy can still be detected on magnetic resonance imaging (MRI) in stable natalizumab-treated patients.^3^ What remains unclear, if there is a specific dose of natalizumab required to ensure optimal slowing of neurodegeneration in MS.

There is an ongoing tendency to personalize treatments to lower treatment burden, risks such as progressive multifocal leukoencephalopathy (PML), and healthcare costs.^5^ Currently, the most adopted treatment strategy for natalizumab is extended interval dosing (EID), where the standard treatment interval of every 4 weeks is prolonged, resulting in lower natalizumab drug trough concentrations and a lower risk of PML in John Cunningham Virus (JCV) positive patients.^5^ The influence of EID and natalizumab drug concentrations on neuroprotective effects of natalizumab is important to consider.

The objectives of this study were to explore whether EID and lower natalizumab drug concentrations were associated with increased development of brain atrophy over time compared to standard interval dosing (SID).

## Materials and methods

### Study design and participants

This was a monocenter retrospective cohort study conducted at the MS center Amsterdam. Patients treated with natalizumab with a diagnosis of RRMS according to the 2017 McDonald criteria, at least two available MRI scans with a minimum follow-up of 2 years, and availability of serum samples were eligible for inclusion. Participants on EID were on a natalizumab treatment interval of ⩾5 weeks in two previous prospective studies.^6,7^ Participants on SID received natalizumab every 4 weeks. All participants provided written informed consent for the collection of blood samples from the Amsterdam UMC MS biobank and collection of data from the electronic patient files. Approval was obtained from the Institutional Ethics Committees.

### Serum natalizumab drug trough concentration

For participants on EID, all available results of natalizumab drug trough concentrations measured in two prospective studies on EID of natalizumab were used.^6,7^ Blood samples were collected before every treatment (range treatment interval 5–7 weeks, *n* = 27)^6^ or every 3–6 months depending on the treatment interval (range treatment interval 5–9 weeks, *n* = 5).^7^ For participants on SID, natalizumab drug concentrations are usually stable within persons on a regular treatment interval.^5^ We therefore selected blood samples of Year 1, Year 3, and last follow-up after the start of natalizumab from our MS Biobank. Natalizumab concentrations were measured by ELISA at Sanquin, The Netherlands, similar to previous trials.^6,7^

### MRI

All available 3D-Fluid Attenuated Inversion Recovery (FLAIR) MRI scans performed during regular clinical follow-up were collected. Only MRI time points at least 1 year after treatment initiation until last follow-up were included in the analyses to correct for pseudo-atrophy.^8^ Brain segmentation was performed with SynthSeg^+^ software, provided in the neuroimaging package FreeSurfer v7.3.2. Total intracranial volume (ICV) and whole brain, ventricular and thalamic volume were selected for further analysis.

### Statistical analyses

The longitudinal associations between study group, mean natalizumab drug trough concentration, and brain volume measures (log-transformed) were investigated with linear mixed-effect models, with study group or concentration, time, and the interaction with time included as fixed effects, and subjects and time as random effects. From the resulting β coefficients for the interaction between time and treatment group, the average yearly percentage change in volume was calculated. All analyses were corrected for age, sex, body mass index (BMI), disease duration, ICV per time point, and type of MRI scanner. A *p*-value less than 0.05 (two-tailed) was considered statistically significant. Analyses were performed in R (version 4.0.3).

## Results

In total, 82 participants were included. Median disease duration was 7.8 years and median radiological follow-up from first MRI was 3.2 years. Participants on EID were older and had a longer disease duration than participants on SID (Table 1).

**Table 1.** Participant characteristics.

	SID (*N* = 50)	EID (*N* = 32)	Total (*N* = 82)	*p* value
Baseline				
Age, years	34.4 ± 9.5	43.6 ± 10.4	38 ± 10.8	**<0.001**
Sex (female), *N* (%)	35 (70)	22 (68.8)	57 (69.5)	0.91
Body weight, kg	78.2 ± 16.2	74.8 ± 11.8	76.7 ± 14.5	0.32
Body mass index, kg/m^2^	25.5 ± 5	24.1 ± 3.9	24.8 ± 4.6	0.21
EDSS score	3.0 (2.5–5.5)	4.3 (3.0–6.0)	3.5 (2.5–6.0)	0.11
JCV status positive, *N* (%)	3 (6)	15 (47)	18 (22)	**<0.001**
JCV index in JCV positive participants	1.0 ± 0.9	1.2 ± 1.1	1.2 ± 1.1	0.91
Time between date of disease onset and baseline^a^, years	7.2 (4–12)	16 (9.8–21.5)	10.4 (5.5–16.9)	**<0.001**
Time between date of diagnosis and baseline (disease duration)^a^, years	5.4 (2.2–8.7)	12.8 (8.1–17.9)	7.8 (3.6–12.7)	**<0.001**
Time between baseline and first MRI scan^a^, years	1.2 (0.99–1.8)	0.04 (0.00–0.08)	0.98 (0.06–1.4)	**<0.001**
DMT before start NTZ, *N* (%)				0.78^b^
None	9 (18)	5 (15.6)	14 (17.1)	
Dimethylfumaric acid	1 (2)	3 (9.4)	4 (4.9)	
Fingolimod	2 (4)	–	2 (2.4)	
Glatiramer acetate	16 (32)	11 (34.4)	27 (32.9)	
Interferons	22 (44)	12 (37.5)	32 (39.1)	
Teriflunomide	–	1 (3.1)	1 (1.2)	
Whole brain^c^	0.798 (0.777–0.813)	0.787 (0.762–0.808)	0.793 (0.766–0.810)	0.17
Ventricles^c^	0.019 (0.014–0.023)	0.020 (0.012–0.034)	0.019 (0.013–0.026)	0.52
Thalamus^c^	0.010 (0.009–0.010)	0.009 (0.009–0.010)	0.009 (0.009–0.010)	0.59
Follow-up				
MRI, number	6 (4–8)	8 (6.3–12.8)	7 (5–9.3)	**0.002**
Duration of MRI follow-up, years	3.4 (2.6–5.1)	3.1 (2.2–3.7)	3.2 (2.6–3.9)	0.055
MRI scanner type, *n* (%)	*n* = 363	*n* = 298	*n* = 661	**<0.001**
Siemens Avanto 1.5T	18 (5.0)	11 (3.7)	29 (4.3)	
GE Discovery MR750 3.0T	23 (6.3)	11 (3.7)	35 (5.2)	
Philips Ingenuity 3.0T	7 (1.9)	4 (1.3)	11 (1.6)	
Siemens Magnetom Sola 1.5T	6 (1.7)	15 (5.0)	23 (3.4)	
Siemens Magnetom Vida 3.0T	16 (4.4)	57 (19.1)	76 (11.2)	
GE Signa HDxt 1.5T	187 (51.5)	183 (61.4)	379 (55.8)	
GE Signa HDxt 3.0T	9 (2.5)	–	9 (1.3)	
Siemens Sonata 1.5T	22 (6.1)	–	22 (3.2)	
Toshiba Titan 3.0T	75 (20.7)	17 (5.7)	95 (14.0)	
Blood samples, number	3 (3–3)	15 (12–17)	3 (3–14)	**<0.001**
Serum NTZ concentration, μg/mL	18.4 (10.8–28.9)	13.1 (11.1–17)	15.1 (11–23.3)	**0.017**

SID: standard interval dosing; EID: extended interval dosing; EDSS: Expanded Status Disability Scale; JCV: John Cunningham Virus; MR: magnetic resonance imaging; DMT: disease-modifying therapy; NTZ: natalizumab; ICV: intracranial volume.

Values are presented as mean values with standard deviation (±), median values with interquartile ranges or frequencies with percentages (%). Values were compared between groups using the chi-square test for categorical variables, the *t*-test for normally and the Mann–Whitney *U* test for non-normally distributed continuous variables. A *p*-value<0.05 (two-tailed) was considered statistically significant.

SID group: 363 available MRI scans. Values are presented as fraction of total ICV. For cross-sectional comparisons of brain volume measures at baseline between groups, volumes were normalized for head size by dividing the tissue volume by the ICV.

a Baseline represents the start of natalizumab therapy on either EID or SID.

b For statistical testing, DMT before the start of NTZ was dichotomized into previous DMT yes/no.

c Normalized MRI measures at first MRI (EID: start of EID; SID: 1 year after the start of natalizumab). EID group: 298 available MRI scans.

**Figure 1.**
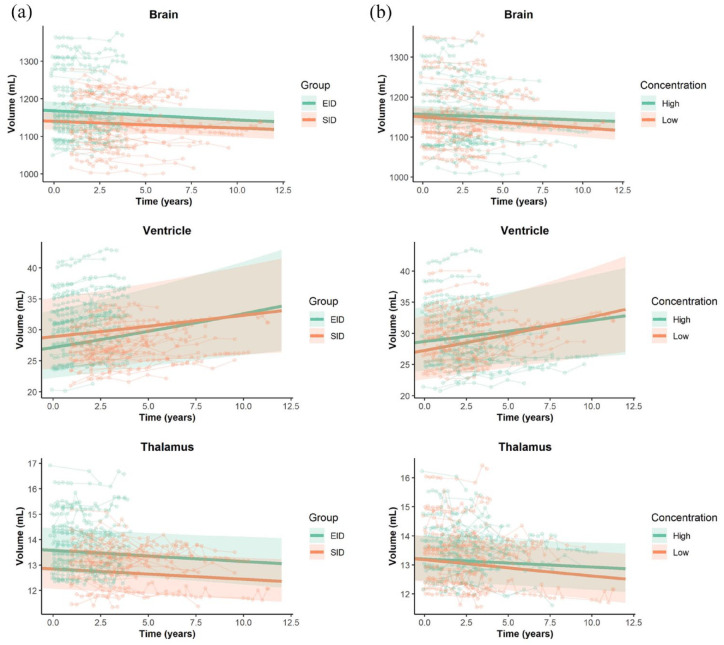
(a) Brain volume measures over time since start of EID and SID (b) or with high or low natalizumab drug trough concentrations. (a) The longitudinal associations between study group and brain volume measures (log-transformed) were investigated with linear mixed-effect models. Whole brain (group × time): Std. β −0.014, Std. error 0.017, *p* = 0.42; Ventricles: Std. β 0.035, Std. error 0.030, *p* = 0.24; Thalamus: Std. β −0.001, Std. error 0.031, *p* = 0.97. EID = extended interval dosing (depicted in green); SID = standard interval dosing (depicted in orange). (b) Natalizumab drug concentration was divided with a median split into low (0–14.4 μg/mL, depicted in orange) and high (14.4–67.0 μg/mL, depicted in green). The longitudinal associations between natalizumab concentration and brain volume measures (log-transformed) were investigated with linear mixed-effect models. Whole brain (low concentration × time): Std. β −0.026, Std. error 0.013, *p* = 0.054; Ventricles: Std. β 0.028, Std. error 0.024, *p* = 0.24; Thalamus: Std. β −0.031, Std. error 0.022, *p* = 0.18. When dividing natalizumab concentration into quartiles (Q2–Q4 compared to Q1), there were no significant associations (Q1 0–10.6 μg/mL, *n* = 17; Q2 10.6–14.4 μg/mL, *n* = 19; Q3 14.4–20.6 μg/mL, *n* = 23; Q4 20.6–67.0 μg/mL, *n* = 23).

Overall, whole brain and thalamic volume decreased over time, while ventricular volume increased over time (Figure 1(a)). We found no differences in volume changes between EID and SID for whole brain (−0.21% vs −0.16%, *p* = 0.42), ventricular (1.84% vs 1.13%, *p* = 0.24), and thalamic (−0.32% vs −0.32%, *p* = 0.97) volume changes after correcting for confounders.

We found no associations between mean natalizumab drug trough concentration and whole brain, ventricular, and thalamic volume changes after correcting for confounders. When dividing natalizumab drug concentration with a median split into low (0–14.4 µg/mL) and high (14.4–67.0 µg/mL), there was a trend towards a higher whole-brain atrophy rate over time in the low group compared to the high group (Figure 1(b)). When dividing natalizumab concentration into quartiles (Q2–Q4 compared to Q1), there were no significant associations.

## Discussion

In our study, we found no clear evidence that EID or lower natalizumab drug trough concentrations are associated with the development of brain atrophy over time in patients with RRMS.

Our results are reassuring, as EID can be beneficial to reduce treatment burden, side effects such as PML in JCV-positive patients, and healthcare costs.^5^ EID of every 6 weeks was equally effective compared to SID in the NOVA trial.^9^ In line with our study, similar results regarding EID and brain atrophy were reported in an exploratory analysis of MRI endpoints of the NOVA trial compared to SID.^10^

In addition, we also found no evidence of an effect of lower natalizumab concentrations on brain atrophy. There was a trend toward a higher whole-brain atrophy rate over time in participants with lower natalizumab drug trough concentrations after applying a median split. We therefore evaluated the highest versus the lowest quartile of natalizumab concentration and found no association with brain atrophy rate over time. In a study with higher doses of natalizumab, no data on brain atrophy were reported, and in trials studying EID of natalizumab, no data on drug concentrations were disclosed.^1,5,10^ It would be of high interest to reassess the results on brain atrophy of these previous trials with regard to natalizumab drug concentrations in a larger cohort.

Limitations of our study include the retrospective design and small sample size, especially when dividing groups into quartiles. The heterogeneity in used MRI scanners and acquisition protocols might bias the assessed brain volume measures, although we corrected for scanner type. The median number of available MRI scans per participant in the EID group (*n* = 8) was higher than in the SID group (*n* = 6, *p* = 0.002). However, follow-up duration was comparable between groups (3.1 and 3.4 years, respectively). Our follow-up of 3.2 years remains relatively short to consider development of brain atrophy and neurodegeneration.

In conclusion, we explored the relation between EID of natalizumab, drug trough concentrations, and brain atrophy measures over time and found no significant associations. Although these results should be confirmed in a larger cohort, we found no clear evidence that EID has a negative impact on brain atrophy rate.
